# Rapid Detection of Black Pepper Adulteration with Endogenous and Exogenous Materials: Assessment of Benchtop and Handheld Infrared Spectrometers

**DOI:** 10.3390/foods15040754

**Published:** 2026-02-19

**Authors:** Paul Rentz, Alina Mihailova, Horacio Heinzen, Martine Bergaentzlé, Elisa Ruhland, Marivil D. Islam, Islam Hamed, Christina Vlachou, Simon Kelly, Said Ennahar, Dalal Werner

**Affiliations:** 1 Chimie Analytique des Molécules Bioactives et Pharmacognosie, Université de Strasbourg, Centre National de la Recherche Scientifique, Institut Pluridisciplinaire Hubert Curien UMR 7178, 67000 Strasbourg, France; p.rentz@aerial-crt.com (P.R.); bergaent@unistra.fr (M.B.);; 2 Aerial, 250 Rue Laurent Fries, Parc d’Innovation, 67400 Illkirch, France; 3 Food Safety and Control Laboratory, Joint FAO/IAEA Centre of Nuclear Techniques in Food and Agriculture, Department of Nuclear Sciences and Applications, International Atomic Energy Agency, Vienna International Centre, P.O. Box 100, 1400 Vienna, Austriaislam.hamed@gmx.at (I.H.);; 4 Grupo de Análisis de Compuestos Traza, Cátedra de Farmacognosia y Productos Naturales, Departamento de Química Orgánica, Facultad de Química, Universidad de la República, General Flores 2124, Montevideo 11800, Uruguay

**Keywords:** black pepper, authenticity, adulteration, mid-infrared spectroscopy, near-infrared spectroscopy, rapid screening, chemometrics

## Abstract

Black pepper is the most widely used spice crop globally and has significant economic value, making it a target for economically motivated adulteration. A wide range of organic and inorganic bulking materials has been used as adulterants in black pepper. Development of rapid non-targeted screening methods for use at different stages of the black pepper supply chain is extremely important for the identification and prevention of evolving fraudulent practices. This study has assessed the potential of benchtop Fourier Transform infrared with attenuated total reflectance (FTIR-ATR), benchtop Fourier Transform near-infrared (FT-NIR), and two handheld NIR spectrometers, coupled with chemometrics, for the discrimination of black pepper (*Piper nigrum*), pepper from other species and genera (non-*Piper nigrum*) and a broad range (*n* = 27) of endogenous and exogenous adulterants. Spiked samples were prepared to imitate pepper adulteration with seven different adulterants at five levels of adulteration (5%, 25%, 50%, 75%, 95% *w*/*w*). Orthogonal partial least squares discriminant analysis (OPLS-DA) achieved 100% total prediction accuracy for both FTIR-ATR and FT-NIR in differentiating authentic *Piper nigrum* and adulterant samples. The handheld microNIR 1700ES resulted in a 91.30% correct classification rate, while the SCiO model achieved 86.96% prediction accuracy. Detection of black pepper adulteration with multiple adulterants was performed using data-driven soft independent modelling of class analogy (DD-SIMCA). The highest performance of the DD-SIMCA model was achieved by FTIR-ATR (100% sensitivity and 100% specificity) followed by FT-NIR (98% sensitivity and 99% specificity). The handheld microNIR 1700ES resulted in 95% sensitivity and 90% specificity. This study demonstrated that FTIR-ATR and FT-NIR, coupled with DD-SIMCA, can effectively detect black pepper adulteration with multiple endogenous and exogenous adulterants. The handheld NIR (microNIR1700ES) clearly demonstrated the potential for rapid and effective verification of *Piper nigrum* authenticity outside the laboratory.

## 1. Introduction

Black pepper, often referred to as the “King of Spices” and “Black Gold”, is the most widely used and, economically, the most important spice crop worldwide [1]. It is obtained from the fruits of *Piper nigrum* L., a perennial climbing vine that is native to the evergreen forests of southwestern India. In addition to its highly valued culinary uses, black pepper is also used in traditional medicine due to its anti-inflammatory and antioxidant properties [2]. Over the past decade, the global consumption and production of black pepper have been steadily increasing with over 812,600 tons produced in 2022. The leading producers of black pepper are Vietnam, Indonesia, Brazil, India, and Burkina Faso [3].

Many varieties of pepper, which are cultivated worldwide, have different yields, organoleptic properties and pungency levels [2,4,5]. In addition, different ripening stages and processing steps are used during the production process yielding black, white, green, and red pepper. Black pepper is obtained from fully mature peppercorns before they are fully ripe [6,7], which are then harvested, separated from the stalks and dehydrated, usually by drying in the sun, until they turn black. On the other hand, white pepper is obtained from the fully ripe (red) peppercorns that are soaked in water to ferment and soften the skin, which is then removed leaving the inner, pale seed to be sun-dried [6,7]. Green pepper is obtained from unripe green berries, similar to black, but preserved in brine, vinegar, or freeze-dried to prevent browning [7]. Red *Piper nigrum* is harvested when the berries are fully mature and red, then air-dried without removing the skin; this type of pepper is not very widely used and is often more expensive due to longer production times [8,9].

The economic importance and growing prices of black pepper, along with its complex supply chain, make this spice crop a target for economically motivated adulteration. Adulteration describes the process of extending or completely substituting black pepper with cheaper fruits or seeds of other species, and many such incidents have been reported [4,10]. Adulterants in ground black pepper can be classified as endogenous (originating from *Piper nigrum* plant), organic exogenous and mineral exogenous [4]. Endogenous adulterants include stem and chaff of black pepper, pinheads (small under-developed berries of *Piper nigrum*), light berries (berries of *Piper nigrum* that have reached a seemingly normal stage of development but are devoid of seed), and black pepper spent (waste or by-product from the black pepper oil extraction process) [4,11,12]. Organic exogenous adulterants can include a wide range of cheaper plant-based bulking materials such as papaya seeds (commonly used due to their structural resemblance), chili, millet, buckwheat, wheat and corn flour, beans, Juniper berry, starch [4,10,11,13,14,15,16,17,18,19,20,21]. Mineral exogenous adulterants can include clay, gypsum, chalk, sand, and gravel [4,14]. A recent example of ground black pepper fraud was reported by the Government of Canada in June 2024 when ground pepper containing sand and dust was recalled in Ontario [22].

The complexity of the supply chain of black pepper and the multitude of potential adulterants require rapid analytical approaches that can verify the authenticity at different stages of production and supply. Several complementary techniques have been investigated for this purpose, including DNA metabarcoding [23], direct analysis in real-time mass spectrometry (DART-MS) [11,24] and proton nuclear magnetic resonance (^1^H NMR) spectroscopy [25]. DNA metabarcoding is a highly sensitive method that can detect botanical adulterants at low levels and identify multiple species in a single, complex, or processed sample [23]. However, it cannot detect mineral adulterants and requires sophisticated bioinformatics and data analysis tools, as well as costly, specialized equipment. DART-MS offers very rapid analysis with minimal sample preparation and high sensitivity, making it well suited for high-throughput screening [11,24]. However, the absence of chromatographic separation can result in ion competition effects, potentially affecting detection selectivity, and the instrumentation remains relatively expensive. ^1^H NMR spectroscopy allows the identification of major metabolites and global compositional differences [25]; however, due to the complex nature of pepper extracts, there can be a significant signal overlap, which can complicate interpretation, and, furthermore, NMR spectrometers are costly and require significant investment in maintenance.

In contrast, vibrational spectroscopy has several distinct advantages and offers non-destructive, relatively low-cost analysis that requires minimal or no sample preparation, offers rapid turnaround time for results and does not generate chemical waste. Along with chemometric modelling, infrared spectroscopy techniques generate fingerprints of authentic samples and rapid monitoring of subtle compositional changes that can highlight atypical (fraudulent) samples. Consequently, the recent development and growing popularity of handheld NIR spectrometers now extend this capability beyond the laboratory, enabling rapid authenticity screening at different stages of the supply chain. Such rapid real-time monitoring may facilitate effective intervention that would allow timely detection and removal of suspect samples from the market, rejection of stocks or shipments, and prevention of distribution, sale and consumption, therefore mitigating possible safety issues with potentially unsafe adulterants, costly recalls and brand reputational damage [26].

Several studies reported successful applications of mid- and near-infrared spectroscopy [11,21,27,28,29] and spectral imaging [4,10] for the detection of adulteration in black pepper. Due to the nature of food fraud and fluctuating commodity prices, it is often difficult to predict which adulterants may be added to black pepper. Therefore, a non-targeted one-class model, which can detect a broad range of adulterant materials, for the verification of typical (*Piper nigrum*) and atypical (adulterated *Piper nigrum*) samples, is very useful. The Data-Driven Soft Independent Modelling of Class Analogy (DD-SIMCA) approach is particularly well suited for this purpose. Unlike (O)PLS-DA, which establishes decision boundaries based on known adulterants, DD-SIMCA builds a model exclusively from authentic *Piper nigrum* samples and identifies those that deviate beyond statistically defined limits. This property makes it especially valuable for routine quality control, where the nature of potential adulterants is often unknown prior to analysis. By modelling the genuine class, DD-SIMCA provides a universal and powerful tool for the detection of both known and unknown adulterants. Previous studies on other food matrices have demonstrated the efficiency of coupling infrared spectroscopy with DD-SIMCA [30,31].

In this context, we propose a versatile analytical strategy based on DD-SIMCA to improve the detection of adulteration and support the authenticity verification of black pepper. This study systematically evaluates and compares four infrared (IR) spectroscopic techniques—benchtop Fourier Transform infrared spectroscopy with attenuated total reflectance (FTIR-ATR), benchtop Fourier Transform near-infrared spectroscopy (FT-NIR), and two miniaturized handheld NIR spectrometers—for the discrimination of *Piper nigrum* and non-*Piper nigrum* samples as well as a broad range of adulterants using a one-class modelling approach. A diverse set of endogenous and exogenous adulterants was considered in order to reflect realistic adulteration scenarios encountered in the food supply chain. In addition, the discrimination of black, white, and red *Piper nigrum* was investigated. To our knowledge, this is the first study to jointly assess benchtop and portable IR spectroscopic platforms combined with DD-SIMCA for black pepper authentication across multiple adulterant classes. The applicability of these approaches for rapid authenticity verification is discussed, with particular emphasis on the potential of handheld NIR sensors for point-of-contact testing of *Piper nigrum* outside the laboratory.

## 2. Materials and Methods

### 2.1. Samples

A total of 40 authentic black pepper (*Piper nigrum*) samples were used in this study. *Piper nigrum* samples originated from India (*n* = 8), Vietnam (*n* = 5), Madagascar (*n* = 3), Tanzania (*n* = 1), Brazil (*n* = 5), Malaysia (*n* = 2), Cambodia (*n* = 5), Cameroon (*n* = 4), Indonesia (*n* = 1), Sri-Lanka (*n* = 1), Vanuatu (*n* = 1), Sao Tome (*n* = 1), Laos (*n* = 1), and Colombia (*n* = 1); one sample (*n* = 1) was an authentic multi-origin mix. In addition, 9 non-*Piper nigrum* samples were used, comprising *Piper guineense* (*n* = 1, Congo), *Piper borbonense* (*n* = 1, Madagascar), *Piper retrofractum* (*n* = 3, Cambodia), *Zanthoxylum armatum* (*n* = 1, Nepal), *Zanthoxylum piperitum* (*n* = 2, China) and *Xylopia aethiopica* (*n* = 1, Senegal). Furthermore, a total of 27 endogenous and exogenous adulterants were used. Endogenous adulterants comprised black pepper processing by-products: black pepper spent and pinheads. Organic exogenous adulterants included papaya seeds, red chili pepper seeds, red chili pepper flesh, walnut shells, hazelnut shells, millet, white sesame, white beans, black lentils, wheat flour, corn flour, white rice flour, brown rice flour, buckwheat flour, potato starch, corn starch, cayenne pepper, olive pomace, black mustard, yellow mustard seeds, hemp seeds, flaxseed. Mineral exogenous adulterants included green clay. Detailed sample information is provided in the Supplementary Information, Table S1.

Samples were stored at −20 °C in the dark. Prior to spectroscopic analyses, samples were cryo-ground to a fine powder using an A11 basic grinder (IKA-Werke, Staufen, Germany).

In addition, 35 adulterated *Piper nigrum* samples were prepared. For the simulated adulteration experiment, *Piper nigrum* from Madagascar was adulterated with 7 selected endogenous and exogenous adulterants: pinheads, black pepper spent, papaya seeds, millet, sesame, black mustard seeds, and corn starch. The following simulated adulteration concentration ‘levels’ were used: 5%, 25%, 50%, 75%, and 95% (*w*/*w*).

### 2.2. Infrared Spectroscopy Analysis

#### 2.2.1. FTIR-ATR Analysis

FTIR-ATR analysis was performed using benchtop Nicolet iS50 FT-IR spectrometer with attenuated total reflectance (ATR) accessory Smart iTX (Thermo Scientific, Waltham, MA, USA). The analysis was carried out using the following settings: spectral range from 4000 to 525 cm^−1^; resolution: 4 cm^−1^; number of scans per sample: 32; number of background scans: 32. Ten replicate samples (approx. 5 mg) were analyzed and spectra averaged.

#### 2.2.2. FT-NIR Analysis

FT-NIR analysis was performed in reflection mode using benchtop MPA II FT-NIR spectrometer (Bruker Optics, Ettlingen, Germany). The analysis was carried out using the following settings: spectral range from 11,550 to 3950 cm^−1^; resolution: 16 cm^−1^; number of scans per sample: 64; number of background scans: 64. Three replicate samples (approx. 3 g) were analyzed and spectra averaged.

#### 2.2.3. Handheld NIR Analysis

Two handheld NIR spectrometers were used in this study: microNIR 1700ES (VIAVI Solutions Inc., San Jose, CA, USA) and SCiO (Consumer Physics, San Francisco, CA, USA). Six (*n* = 6) replicate samples (approx. 3 g) were analyzed using each handheld device and spectra averaged. The following settings were used for the NIR analysis with the microNIR 1700ES: spectral range from 10,515 to 6057 cm^−1^ (951 to 1651 nm); resolution: 6 nm; number of scans per sample: 100. The following settings were used for the NIR analysis with SCiO: spectral range from 13,514 to 9346 cm^−1^ (740 to 1070 nm); resolution: 1 nm; and number of scans per sample: 1.

### 2.3. Chemometric Analysis

Spectral data pre-processing and chemometrics analysis were performed using SIMCA software, version 16.0.2 (Sartorius Stedim Biotech GmbH, Göttingen, Germany) and the Chemometrics 2.0 Add-in for Excel (CAFE) [32]. Different spectral pre-processing algorithms were applied and compared for each IR spectroscopy technique to select the best pre-processing approach based on the chemometric model performance. The following pre-processing methods were used: data centering (all data), 1st derivative (FTIR-ATR and FT-NIR), 2nd derivative (microNIR 1700ES) and standard normal variate (SCiO).

Orthogonal partial least squares discriminant analysis (OPLS-DA) was used to discriminate (i) *Piper nigrum* and adulterant samples, (ii) *Piper nigrum* and non-*Piper nigrum*, and (iii) black, white and red *Piper nigrum*. The performance of the OPLS-DA models was assessed using the cumulative goodness of fit (R2 (cum)) and predictability (Q2 (cum)) values. R2 describes how well the data are explained by the model, while Q2 describes the predictive ability of the model after internal seven-fold cross-validation. For the discrimination of *Piper nigrum* and the adulterants, the full dataset was split into a training dataset (*n* = 44), which was used for the generation of the OPLS-DA model, and a test dataset (*n* = 23), which was used for validation. The model performance was assessed using the correct classification rate for the test dataset. The NIR absorption bands, which were significant for the differentiation of the *Piper nigrum* and the adulterants, were examined using the averaged spectral data and Variable Importance in Projection (VIP) scores (VIP score > 1) from the generated OPLS-DA models.

**Figure 1:**
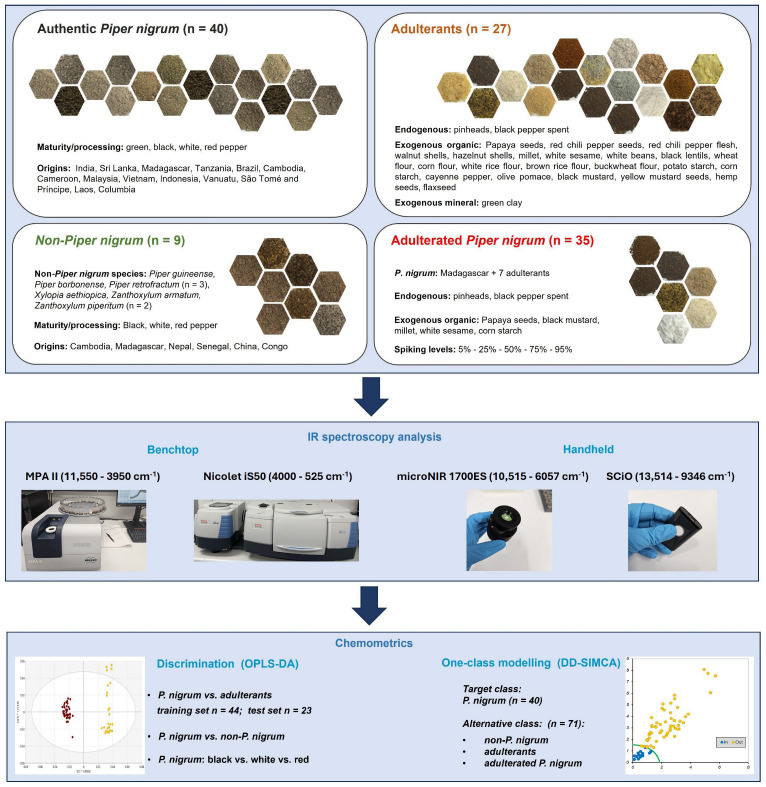
The workflow of this study.

Additionally, data-driven soft independent modelling of class analogy (DD-SIMCA) [33] was performed using the Chemometrics for Excel Add-In (CAFE) [32]. DD-SIMCA is a supervised one-class classification method based on principal component analysis (PCA) that is widely used for the verification of authenticity in the framework of non-targeted analysis. To develop a DD-SIMCA classification model, authentic *Piper nigrum* samples (*n* = 40) were defined as the target class with 30% of the samples defined as an internal test set. Non-*Piper nigrum* (*n* = 9), adulterants (*n* = 27) and adulterated *Piper nigrum* samples (*n* = 35) were assigned to the alternative class (*n* = 71). Performance of the DD-SIMCA models was expressed in terms of sensitivity for a given type I error rate expressed as a percentage, alpha (α) and specificity with the resulting type II error rate expressed as a percentage, beta (β) [32]. The workflow of this study is shown in Figure 1.

## 3. Results and Discussion

### 3.1. IR Spectral Profiles of Piper nigrum, Non-Piper nigrum, Endogenous and Exogenous Adulterants

**Figure 2:**
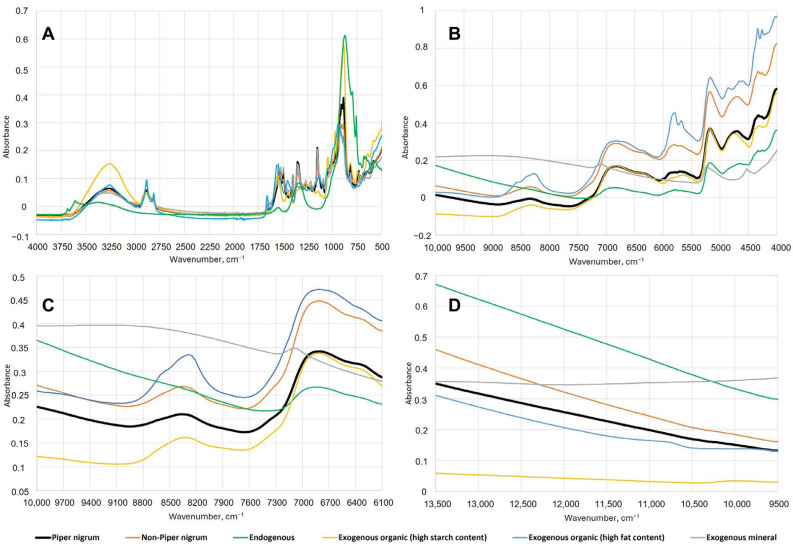
Raw averaged IR spectra of *Piper nigrum*, non-*Piper nigrum*, endogenous adulterants, exogenous organic adulterants with high starch content, exogenous organic adulterants with high fat content and exogenous mineral adulterants, acquired using benchtop Nicolet iS50 FTIR-ATR (**A**), benchtop MPA II FT-NIR (**B**), handheld microNIR 1700ES (**C**) and handheld SCiO (**D**).

Significant differences in the spectra of *Piper nigrum*, non-*Piper nigrum* and each of the endogenous and exogenous adulterants were observed using mid- and near-infrared spectroscopy. For a better visual presentation of the spectral data, the spectra were grouped and averaged as follows: (i) *Piper nigrum*, (ii) non-*Piper nigrum*, (iii) endogenous adulterants (black pepper spent and pinheads), (iv) exogenous organic adulterants with high starch content (wheat flour, white rice flour, brown rice flour, buckwheat flour, corn flour, corn starch, potato starch, millet, white beans, black lentils), (v) exogenous organic adulterants with high fat content (sesame seeds, hemp seeds, flaxseeds, yellow mustard seeds, black mustard seeds, red chili pepper seeds, olive pomace), (vi) exogenous mineral adulterants (green clay). Figure 2 presents the mean raw absorbance spectra of the above-mentioned sample groups acquired using benchtop FTIR-ATR and FT-NIR, and two handheld NIR spectrometers.

The mid-infrared spectra (Figure 2A) show important differences in the range between 1700 and 600 cm^−1^ as well as between 3750 and 2740 cm^−1^. The broad absorption band centred at around 3280 cm^−1^ corresponds to the stretching vibration of hydroxyl (−OH) groups, which can be mainly attributed to the presence of water and high starch content. The 3000–2750 cm^−1^ spectral region, consisting of two peaks, corresponds to C−H asymmetric and symmetric stretching vibrations that are associated with lipids [27,34]. The absorbance in this region is much higher for the exogenous adulterant samples with high fat content. The fingerprint region is found between 1700 and 600 cm^−1^ and has a number of bands associated with the CH_2_, CH_3_, C−C, C=C, C−O, C=O, C−N and C−O−C vibrations [34,35,36]. These vibrations can be primarily associated with piperine and the major differences in the lipid, protein and carbohydrate profiles between *Piper nigrum*, non-*Piper nigrum*, endogenous and exogenous adulterants. Piperine is one of the major non-volatile alkaloids present in black pepper, and it is responsible for its pungency [1,8]. The presence of piperine has been reported to be associated with specific signals at 1633 cm^−1^, 1580 cm^−1^, 1252 cm^−1^ and 996 cm^−1^ [5] and is an important factor for the spectral differences between *Piper nigrum* and adulterant matrices. In addition, the spectral bands between 1100 and 500 cm^−1^ can be attributed to the C−H (trans-) and C−H (cis-) out-of-plane bending vibrations of the constituents of black pepper essential oil. The essential oil, which is responsible for the pungent aroma of black pepper, consists of around 80% monoterpenes (e.g., sabinene, myrcene, α- and β-pinene, limonene, 1,8-cineol, α-phellandrene) and around 20% sesquiterpenes (e.g., α- and β-caryophyllene, humulene) [8,37]. The strong absorption band at around 900 cm^−1^ corresponds to the stretching vibration of exo-methylene (−CH_2_−) of terpene compounds in black pepper [27]. Starch is the predominant constituent of black pepper, ranging from 35 to 40% of its weight [38]. The 1390–875 cm^−1^ spectral region is associated with the absorption bands of starch and cellulose [4] where the main differences between *Piper nigrum* and starchy exogenous adulterants are observed.

The near-infrared spectra show important differences between *Piper nigrum*, non-*Piper nigrum*, endogenous and exogenous adulterants primarily in the spectral region between 9000 and 4000 cm^−1^ (Figure 2B,C). Absorptions of overtones or combinations of fundamental stretching vibrations of functional groups associated with piperine have been reported to be in the following regions: 6200–5300 cm^−1^, which is associated with the 1st overtone of C−H stretching vibrations, and 5000–4000 cm^−1^ associated with C−H stretching and C−H deformation combinations [39]. The 9000–8000 cm^−1^ and 5820–5680 cm^−1^ spectral regions, associated with the second and first overtone of the C−H stretching vibrations, respectively, can be linked to the presence of lipids [40]. As can be seen in Figure 2B, the absorbances at these two regions are much higher for the exogenous adulterant samples with higher fat content. The spectral region between 6200 and 7100 cm^−1^ corresponds to the absorption bands of −OH groups (1st overtone of −OH stretching vibration), which can be associated with water, lignin, starch and other polysaccharides [10,28,41]. In general, the complex nature of NIR spectra, comprising numerous overlapping bands, makes them difficult for interpretation and not particularly useful for identification purposes. However, in combination with chemometric modelling, the differences between *P. nigrum*, endogenous and exogenous adulterants can be exploited for classification.

### 3.2. Discrimination Between Piper nigrum and Adulterants

**Figure 3:**
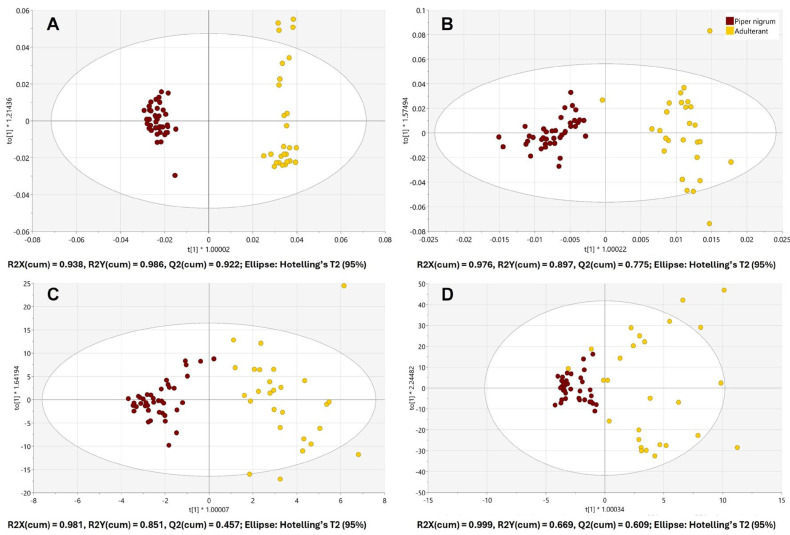
Score plots of the OPLS-DA models for the discrimination of *Piper nigrum* (*n* = 40) and adulterants (*n* = 27) built using the spectral data from benchtop Nicolet iS50 FTIR-ATR (**A**), benchtop MPA II FT-NIR (**B**), handheld microNIR 1700ES (**C**) and handheld SCiO (**D**).

A supervised chemometrics approach, OPLS-DA, was able to discriminate *Piper nigrum* and all 27 adulterants when using the spectral data acquired with benchtop IR spectrometers. The score plots of the 7-fold cross-validated OPLS-DA models, built using benchtop FTIR-ATR and FT-NIR data, are shown in Figure 3A and Figure 3B, respectively. Handheld spectrometers also achieved the separation between *Piper nigrum* and the adulterant class; however, an overlapping of samples from two classes was observed as can be seen in Figure 3C and Figure 3D for microNIR 1700ES and SCiO, respectively.

The goodness of fit (R2X (cum), R2Y (cum)) and the predictive ability (Q2 (cum)) values of the OPLS-DA models as well as the correct classification of the test dataset are shown in Table 1. The highest model performance indicators (R2, Q2) were observed for the OPLS-DA model built using FTIR-ATR data. All four IR spectrometers resulted in 100% correct classification of *Piper nigrum* samples from the test dataset; however, the accuracy of prediction of the adulterant class differed (Table 1). OPLS-DA models built using benchtop FTIR-ATR and FT-NIR data achieved 100% correct classification rate for the adulterant class, which resulted in a total correct classification rate of 100% for both benchtop instruments. These results confirm the efficacy of infrared spectroscopy combined with chemometric analysis in distinguishing authentic *Piper nigrum* from potential adulterants and agree with the findings of several previous studies. As demonstrated by Wilde et al. (2019) [21], infrared spectroscopy is also an effective method of discriminating authentic from adulterated products. These authors employed FTIR and FT-NIR spectroscopy to detect black pepper adulteration with two exogenous (papaya seeds, chili) and three endogenous (black pepper husk, spent and pinheads) materials. The study reported an excellent separation between black pepper and adulterated samples, with a 100% and 98% specificity for the FT-NIR and FTIR, respectively. This was achieved using OPLS-DA and the area under the ROC curve [21]. The results of the present study also support the findings of Hu et al. (2018) [27], who used diffuse reflectance mid-infrared Fourier transform spectroscopy (DRIFTS) for the detection of black pepper adulteration with sorghum and Sichuan pepper, and reported 96% correct classification of the prediction set using a PLS-DA model.

The OPLS-DA model built using data from the handheld microNIR 1700ES resulted in 91.30% total correct classification rate for the test dataset. Considering the small size, portability and a much lower cost of microNIR 1700ES in comparison with benchtop NIR, this high classification result is very promising for the rapid authenticity testing of black pepper outside the laboratory. The discriminative model generated using the SCiO data achieved 86.96% prediction accuracy. Handheld NIR spectrometers differ in the implemented technology, spectral range, analytical performance and cost. SCiO is one of the cheapest commercially available NIR spectrometers; however, the low cost is reflected in its lower technical specification and performance.

The IR absorption bands, which were significant for the differentiation of the *Piper nigrum* and adulterants, were identified using the spectral information and the VIP scores (VIP > 1) from the generated OPLS-DA models. For FTIR-ATR, the significant spectral bands, based on the VIP score, were in the regions between 3000 and 2740 cm^−1^ (C−H asymmetric and symmetric stretching vibrations) and between 1700 and 600 cm^−1^ (CH_2_, CH_3_, C−C, C=C, C−O, C=O, C−N and C−O−C vibrations). For the NIR, the most significant bands were in the region from 8800 to 4000 cm^−1^ (O−H stretch 1st overtone, C−H stretch 1st overtone, C−H stretching and C−H deformation combinations). As discussed in Section 3.1, these mid- and near-infrared spectral regions can primarily be associated with the presence/absence of piperine, differences in the monoterpene, sesquiterpene and fatty acid profiles, as well as starch content, all of which are significant for the discrimination [4,5,10,21,27,28,34,35,36,39,41].

### 3.3. Discrimination Between Piper nigrum and Non-Piper nigrum

**Figure 4:**
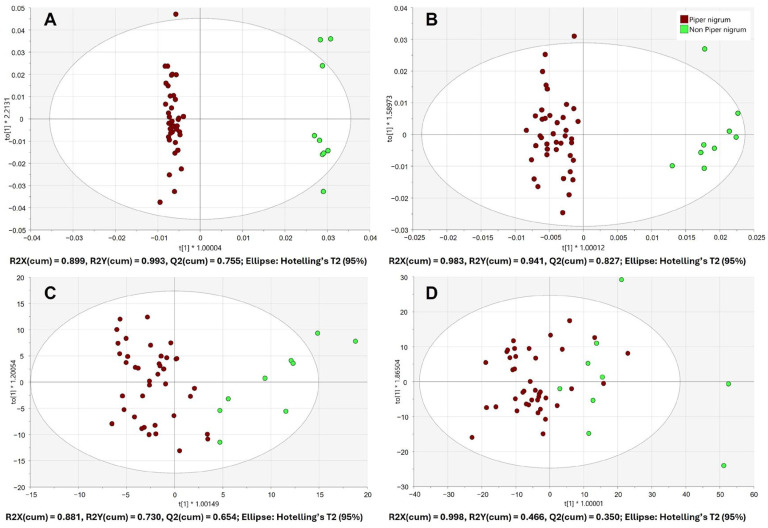
Score plots of the OPLS-DA models for the discrimination of *Piper nigrum* (*n* = 40) and non-*Piper nigrum* (*n* = 9) built using the spectral data from benchtop Nicolet iS50 FTIR-ATR (**A**), benchtop MPA II FT-NIR (**B**), handheld microNIR 1700ES (**C**) and handheld SCiO (**D**).

Additionally, the discrimination between *Piper nigrum* and non-*Piper nigrum* samples was assessed. The score plots of the 7-fold cross-validated OPLS-DA models, built using benchtop and handheld IR spectrometer data, are shown in Figure 4.

Both benchtop spectrometers achieved excellent separation of the two classes (Figure 4A,B). The goodness of fit (R2X (cum), R2Y (cum)) and the predictive ability (Q2 (cum)) values of the OPLS-DA model built using FTIR-ATR data were 0.899, 0.993 and 0.755, respectively. The goodness of fit (R2X (cum), R2Y (cum)) and the predictive ability (Q2 (cum)) values of the OPLS-DA model built using FT-NIR data were 0.983, 0.941 and 0.827, respectively. Some overlap of the *Piper nigrum* and non-*Piper nigrum* classes was observed for the handheld microNIR 1700ES data (Figure 4C). The goodness of fit (R2X (cum), R2Y (cum)) and the predictive ability (Q2 (cum)) values of the OPLS-DA model were 0.881, 0.730 and 0.654, respectively. In contrast, SCiO data did not achieve the separation of the two classes and resulted in low model performance indicators (Q2 < 0.5) as can be seen in Figure 4D. The summary of the OPLS-DA model performance for the discrimination of *Piper nigrum* and non-*Piper nigrum* samples is provided in the Supplementary Information (Table S2).

The IR absorption bands, which were significant for the differentiation of the *Piper nigrum* and non-*Piper nigrum* samples, were identified using the spectral information and the VIP scores (VIP > 1) from the generated OPLS-DA models. The most significant mid-infrared spectral region was between 1680 and 750 cm^−1^ (CH_2_, CH_3_, C−C, C=C, C−O, C=O, C−N, C−O−C vibrations), while the most important near-infrared bands were in the region from 8800 to 4000 cm^−1^ (O−H stretch 1st overtone, C−H stretch 1st overtone, C−H stretching and C−H deformation combinations). These mid- and near-infrared spectral differences between *Piper nigrum* and non-*Piper nigrum* can primarily be associated with different profiles of alkaloids, terpenoids, sterols, polyphenols, monoterpene and sesquiterpene hydrocarbons as well as other components of pepper essential oils that have been reported to differ among different pepper species [8,42,43,44].

### 3.4. Discrimination of Black, White and Red Piper nigrum

**Figure 5:**
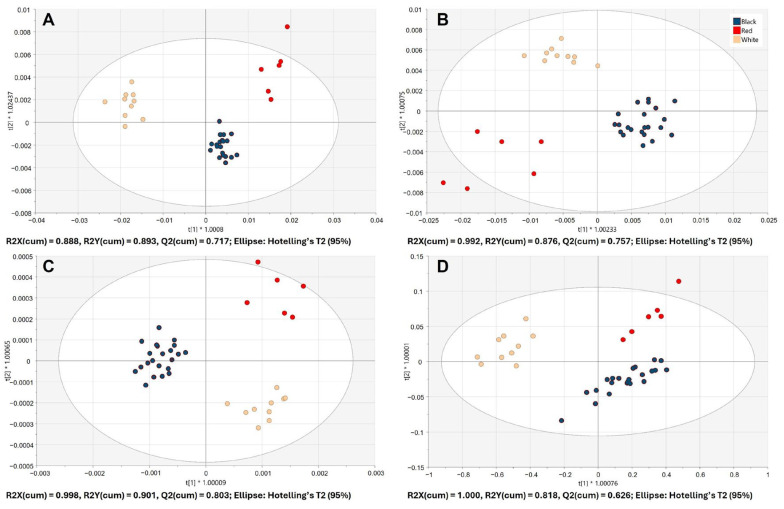
Score plots of the OPLS-DA models for the discrimination of black (*n* = 22), white (*n* = 10) and red (*n* = 6) *Piper nigrum* built using the spectral data from benchtop Nicolet iS50 FTIR-ATR (**A**), benchtop MPA II FT-NIR (**B**), handheld microNIR 1700ES (**C**) and handheld SCiO (**D**).

*Piper nigrum* samples comprised four different types of pepper based on the maturity level and processing, i.e., black, white, red and green pepper. An additional objective was to assess whether the four IR spectroscopy techniques could discriminate between different types of *Piper nigrum*. Green *Piper nigrum* samples were excluded from chemometric modelling due to the low number of available samples, and only the discrimination of black, white and red pepper was performed. The score plots of the 7-fold cross-validated OPLS-DA models, built using benchtop and handheld IR spectrometer data, are shown in Figure 5.

The OPLS-DA models built using the data from all four IR spectrometers achieved excellent discrimination of the three types of pepper without any overlapping of samples from different classes (Figure 5A–D). The predictive ability (Q2 (cum)) values of the seven-fold cross-validated OPLS-DA model built using benchtop FTIR-ATR and FT-NIR, and handheld microNIR 1700ES and SCiO data were 0.717, 0.757, 0.803 and 0.626, respectively. The summary of the OPLS-DA model performance for the discrimination of black, white and red *Piper nigrum* is provided in the Supplementary Information (Table S3).

**Figure 6:**
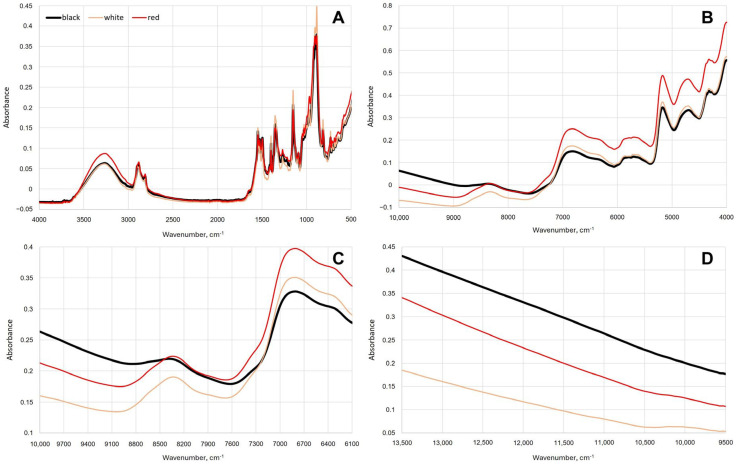
Raw averaged IR spectra of black, white and red *Piper nigrum*, acquired using benchtop Nicolet iS50 FTIR-ATR (**A**), benchtop MPA II FT-NIR (**B**), handheld microNIR 1700ES (**C**) and handheld SCiO (**D**).

Figure 6 presents the mean raw absorbance spectra of the black, white and red *Piper nigrum* acquired using benchtop FTIR-ATR and FT-NIR, and two handheld NIR spectrometers.

The IR absorption bands, which were significant for the differentiation of the black, red and white *Piper nigrum,* were identified using the spectral information and the VIP scores (VIP > 1) from the generated OPLS-DA models. The most significant mid-infrared spectral regions were 1670–560 cm^−1^ (CH_2_, CH_3_, C−C, C=C, C−O, C=O, C−N and C−O−C vibrations) and 2980–2710 cm^−1^ (C−H asymmetric and symmetric stretching vibrations), while in the case of near-infrared spectra, significant bands covered almost the entire NIR spectral region with the most significant bands being between 8900 and 4025 cm^−1^ (O−H stretch 1st overtone, C−H stretch 1st overtone, C−H stretching and C−H deformation combinations). These spectral differences between black, white and red *Piper nigrum* can be associated with differences in starch content and their lipid, alkaloid, monoterpene and sesquiterpene profiles [45]. The bite and pungency in black, white and red peppers is primarily due to the non-volatile alkaloids, piperine and chavicin. Similarly to black *Piper nigrum*, white *Piper nigrum* contains piperine; however, it lacks chavicin that is present in the epicarp, which is removed during the preparation of white pepper [8]. Also, differences in the amount and composition of essential oils extracted from different types of *Piper nigrum* have been reported [8,46]. The content of starch is much higher in white peppers with values ranging from 53 to 58% compared to 35–40% in black pepper [38]. Furthermore, fibre and ash contents are considerably lower in white *Piper nigrum*, as is to be expected because of the removal of the outer pericarp in its preparation [38].

### 3.5. One-Class Modelling (DD-SIMCA) for the Verification of Piper nigrum Authenticity

**Figure 7:**
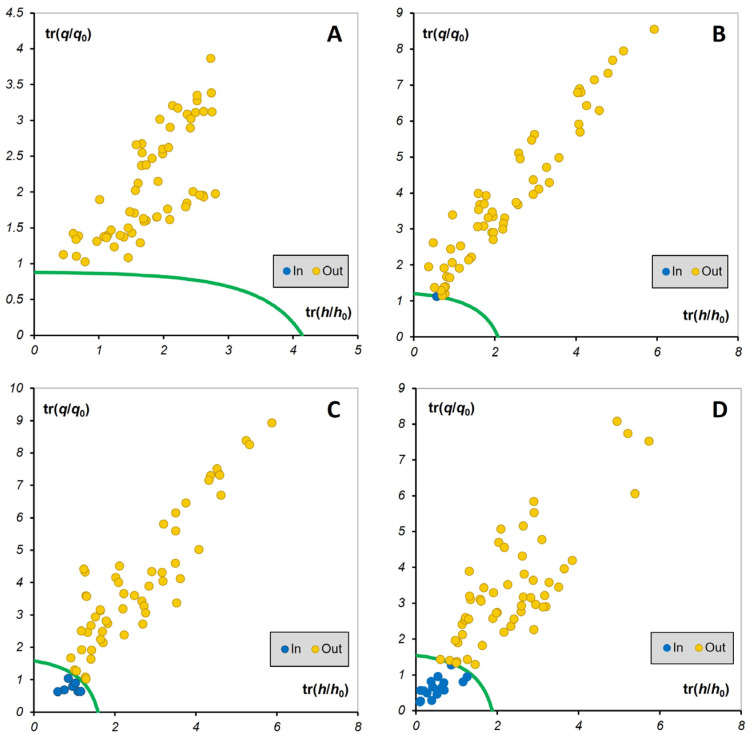
The acceptance plot of the DD-SIMCA model built using the spectral data from benchtop Nicolet iS50 FTIR-ATR (**A**), benchtop MPA II FT-NIR (**B**), handheld microNIR 1700ES (**C**) and handheld SCiO (**D**). The green line indicates the acceptance boundary (95% confidence) for the authentic *Piper nigrum class* (*n* = 40). The yellow circles are the samples from the alternative class (*n* = 71), comprising non-*Piper nigrum* (*n* = 9), adulterants (*n* = 27) and adulterated *Piper nigrum* (*n* = 35), which are correctly classified as non-compliant. The blue circles are the samples from the alternative class that are wrongly assigned to the authentic *Piper nigrum* class. Horizontal axis: tr (h/h_0_) represents the normalized score distance of a sample relative to the DD-SIMCA model. Vertical axis: tr (q/q_0_) represents the normalized orthogonal distance of the sample to the model.

Detection of black pepper adulteration with seven selected endogenous and exogenous adulterants (black pepper spent, pinheads, papaya seeds, millet, sesame, black mustard seeds, corn starch) was assessed using one-class modelling. DD-SIMCA models were generated with the *Piper nigrum* samples (*n* = 40) defined as a target class, while non-*Piper nigrum* samples (*n* = 9), adulterants (*n* = 27) and adulterated *Piper nigrum* samples (*n* = 35) were defined as an alternative class. The sensitivity and specificity of the DD-SIMCA models generated using benchtop FTIR-ATR and FT-NIR and two handheld NIR spectrometers are given in Table 2. Figure 7 presents the acceptance plots of the generated DD-SIMCA models.

The DD-SIMCA model built using the data from benchtop FTIR-ATR showed the highest sensitivity and specificity values of 100%, respectively. All the samples from the alternative class, including adulterated *Piper nigrum*, were correctly assigned by the model as non-authentic (Table 2 and Figure 7A). The DD-SIMCA model built using the data from benchtop FT-NIR also showed high sensitivity and specificity values of 98% and 99%, respectively. All adulterants and non-*Piper nigrum* samples were correctly assigned by the model as non-authentic, while one adulterated *Piper nigrum* sample (*Piper nigrum* + 5% corn starch) was wrongly assigned by the model to the authentic *Piper nigrum* class (Table 2 and Figure 7B). These results are consistent with the findings of Orillo et al. (2019) [10] who achieved a 100% correct classification rate for both the calibration and prediction set using a SIMCA model for the discrimination of black pepper and adulterated pepper with papaya seeds with NIR-hyperspectral imaging (NIR-HSI). DD-SIMCA models built using the data from the handheld NIR spectrometers showed lower model performance compared to that of benchtop spectrometers (Table 2 and Figure 7C,D). The sensitivity of the DD-SIMCA model generated with microNIR 1700ES data was 95%, while its specificity was 90%. All adulterants and non-*Piper nigrum* samples were correctly assigned by the model as non-authentic. Seven adulterated *Piper nigrum* samples were wrongly assigned by the model to the authentic *Piper nigrum* class. These were *Piper nigrum* samples adulterated with black pepper spent, papaya seeds, corn starch, and black mustard at 5% and millet at 5–50%. These findings suggest that rapid detection of adulteration at 25% or above, for most of the tested adulterants, can be successfully achieved using handheld microNIR 1700ES; however, it cannot be considered suitable for the detection of *Piper nigrum* adulteration at 5% or below. This adulteration level, however, can often be considered unlikely for the economically motivated adulteration of black pepper where, depending on the nature of adulterant(s), much higher adulteration levels (up to 40% and sometimes even higher) can be used to balance unlawfully gained profit against risk of detection [11].

The DD-SIMCA model generated using the handheld SCIO spectrometer data resulted in the lowest sensitivity and specificity values of 93% and 77%, respectively. All adulterants and non-*Piper nigrum* samples were correctly assigned by the model as non-authentic; however, 16 adulterated *Piper nigrum* samples, which mostly included *Piper nigrum* adulterated at 5 and 25%, were misclassified as authentic *Piper nigrum*. This is not surprising, considering the limited spectral range of the SCiO spectrometer. Samples that were wrongly assigned by the DD-SIMCA model built using the SCiO data were mostly *Piper nigrum* adulterated at 5–25%. Based on these results and considering the low cost of the SCiO spectrometer, it can be argued that, for some adulterants (e.g., black pepper spent, pinheads, papaya seeds) that have a close resemblance to black pepper and thus can be mixed with black pepper at over 25%, the SCiO may still represent a cost-effective rapid screening tool to detect the suspect samples that would need to be further subjected to confirmatory analysis. This would need to be assessed in a more comprehensive validated adulteration study with *Piper nigrum* samples from multiple origins and production years included in the chemometric model and a wider range of adulterants and a greater number of adulteration steps included in the test set of this chemometric model.

## 4. Conclusions

The development of rapid non-targeted screening methods is extremely important for the identification and prevention of evolving fraudulent practices in the black pepper supply chain. This study assessed the potential of two benchtop and two handheld infrared spectrometers, combined with OPLS-DA and DD-SIMCA as analytical tools for the rapid discrimination of *Piper nigrum*, non-*Piper nigrum* and adulterants as well as the detection of black pepper adulteration, respectively. OPLS-DA was applied to assess the discriminative capability of the different IR platforms under controlled conditions where representative samples of both authentic and non-authentic classes were available and known. This approach is well suited for method comparison and exploratory discrimination tasks. In contrast, DD-SIMCA was employed to simulate realistic routine screening scenarios, where comprehensive knowledge of all possible adulterants is rarely available. One-class modelling is therefore preferable for authenticity verification and fraud detection in practice, as it focuses on defining the boundaries of authentic *Piper nigrum* and identifying deviations from this class.

The OPLS-DA models built using FTIR-ATR and FT-NIR data for the discrimination of authentic *Piper nigrum* and adulterant samples achieved 100% correct classification of the test dataset. Excellent discrimination was also achieved for the *Piper nigrum* and non-*Piper nigrum* classes for both benchtop techniques. The OPLS-DA model built using the data from handheld NIR spectrometers resulted in lower performance compared to that of benchtop instruments as could be expected considering their limited spectral range and small size. These results are in agreement with the findings of Mayr et al. (2021) [47] who compared benchtop FT-NIR and three handheld NIR spectrometers for the quantification of piperine in whole and milled seeds of black pepper and reported a lower performance of the SCIO spectrometer in comparison with benchtop FT-NIR (Büchi NIRFlex N-500) and two other handheld spectrometers (microNIR 2200 and microPHAZIR). However, the obtained results are encouraging. The OPLS-DA model used for the discrimination of *Piper nigrum* and adulterant classes, and built using handheld microNIR 1700ES data, achieved 91.30% correct classification rate for the test dataset, while the model generated using the SCiO data resulted in 86.96% total prediction accuracy for the test set. Discrimination of *Piper nigrum* and non-*Piper nigrum* class was acceptable in the case of microNIR 1700ES but not successful for the SCiO. On the other hand, an excellent discrimination of black, white and red *Piper nigrum* samples was achieved by all four spectrometers.

Detection of black pepper adulteration with seven selected endogenous and exogenous adulterants (black pepper spent, pinheads, papaya seeds, millet, sesame, black mustard seeds, corn starch) was assessed using one-class modelling. The highest performance of the DD-SIMCA model was achieved by FTIR-ATR (100% sensitivity and specificity) followed by FT-NIR (98% sensitivity and 99% specificity). The DD-SIMCA model built using the data from microNIR 1700ES achieved 95% sensitivity and 90% specificity (*Piper nigrum* adulterated with spent, papaya seed and mustard seed at 5% of adulteration misclassified), which shows the high potential of this handheld spectrometer for the detection of black pepper adulteration outside the laboratory. Considering that economically motivated adulteration is generally expected to occur at levels ≥ 10% [21], the misclassifications observed at 5% adulteration indicate that the handheld NIR 1700ES spectrometer can still be considered suitable for rapid pre-screening applications under real-world conditions. The DD-SIMCA model built using the SCiO data showed the lowest sensitivity and specificity values of 93% and 77% (*Piper nigrum* adulterated with millet and corn flour at 50% of adulteration misclassified), respectively.

Even though a limited number of samples were available for this study, the results demonstrate the significant potential of benchtop FTIR-ATR and FT-NIR as well as handheld NIR spectrometers, combined with one-class modelling, as rapid screening tools for the detection of black pepper adulteration with multiple endogenous and exogenous materials. We have assessed a wide range of adulterants, including endogenous, organic and mineral exogenous adulterants, and excellent discrimination was achieved, in particular in the case of benchtop FTIR-ATR and FT-NIR spectroscopy. The main advantages of the above IR spectroscopy techniques include their rapidity, cost-effectiveness, non-destructive nature, and the possibility of omitting sample preparation. In addition, in the case of handheld devices, measurements can be performed outside the laboratory. The observed performance differences between benchtop and handheld NIR spectrometers can be attributed to fundamental hardware differences. Benchtop NIR instruments are characterized by broader spectral coverage, higher spectral resolution, and improved signal-to-noise ratios, resulting in superior discrimination and adulteration detection capabilities. Handheld NIR spectrometers, on the other hand, employ miniaturized optical components and operate over a more limited spectral range, which can reduce sensitivity but offer substantial advantages in portability, cost, and the possibility of on-site measurements. These trade-offs make benchtop instruments more suitable for laboratory-based confirmatory analysis, while handheld devices are particularly attractive for rapid pre-screening applications along the supply chain. This allows for rapid pre-screening to identify and sift uncompliant samples that can be subjected to more expensive and time-consuming techniques for confirmatory analysis. The use of benchtop FTIR-ATR and FT-NIR spectroscopy coupled with OPLS-DA and DD-SIMCA can be considered as a powerful tool for the rapid authenticity testing and detection of adulteration in *Piper nigrum*. Provided that sufficient number of authentic *Piper nigrum*, collected over multiple production years and geographical origins, are included in the model, we could see this as a successful tool for the early rapid detection of black pepper adulteration. To make the testing available at different steps of the supply chain, handheld NIR spectrometers could be employed. Of the two handheld spectrometers tested, microNIR 1700ES showed a higher performance and could be used to test adulteration of *Piper nigrum* above 5%.

Despite the promising results obtained in this study, several research gaps remain. The current limitation of this study is that the simulated adulteration experiments were performed using *Piper nigrum* from a single origin (Madagascar), which may limit model robustness and transferability, as black pepper composition may be influenced by geographical origin, post-harvest processing, and maturity stage. Further work will include model generation and validation using a wider range of authentic *Piper nigrum*, non-*Piper nigrum*, and adulterant samples from multiple regions and production years, as well as a greater number of adulterated *Piper nigrum* samples, taking into account variability due to origin, post-harvest processing (white pepper), and maturity stage at different simulated adulteration concentration levels that are realistic representations of economically motivated adulteration.

In addition, strategies for model transfer and calibration maintenance between benchtop and handheld instruments represent an important area for further research. From a technological perspective, ongoing advances in miniaturized spectrometers, detector sensitivity, and data processing are expected to enhance the performance of portable NIR devices. Finally, the increased adoption of one-class modelling approaches, such as DD-SIMCA, combined with portable spectroscopic tools, is anticipated to play a key role in the development of practical, supply-chain-oriented screening systems for the early detection of food fraud.

## Figures and Tables

**Table 1:** Validation results of the OPLS-DA models for the discrimination of *Piper nigrum* and adulterants generated using the data from benchtop FTIR-ATR and FT-NIR and two handheld NIR spectrometers.

IR Spectrometer	Type of Spectrometer	OPLS-DA (Training Set, *n* = 44)			Discrimination Rate of the Test Set, %		
		R2X (cum)	R2Y (cum)	Q2 (cum)	*Piper nigrum* (*n* = 14)	Adulterants (*n* = 9)	Total (*n* = 23)
Nicolet iS50 FTIR-ATR	benchtop	0.938	0.986	0.922	100	100	100
MPA II FT-NIR	benchtop	0.976	0.897	0.775	100	100	100
microNIR 1700ES	handheld	0.981	0.851	0.457	100	77.78	91.30
SCiO	handheld	0.999	0.669	0.609	100	66.67	86.96

**Table 2:** Performance of the DD-SIMCA models for the authentication of *Piper nigrum* using the data from benchtop FTIR-ATR and FT-NIR, and two handheld NIR spectrometers.

IR Spectrometer	Type of Spectrometer	Sensitivity, % (*Piper nigrum*, *n* = 40)	Specificity, % Alternative Class (*n* = 71)	Samples from Alternative Class Wrongly Assigned as *Piper nigrum*		
				Non-*Piper nigrum*	Adulterants	Adulterated *Piper nigrum*
Nicolet iS50 FTIR-ATR	benchtop	100	100	0	0	0
MPA II FT-NIR	benchtop	98	99	0	0	1
microNIR 1700ES	handheld	95	90	0	0	7
SCiO	handheld	93	77	0	0	16

## Data Availability

The original contributions presented in the study are included in the article/Supplementary Materials. Further inquiries can be directed to the corresponding author.

## Supplementary Materials

The following supporting information can be downloaded at: https://www.mdpi.com/article/10.3390/foods15040754/s1, Table S1: *Piper nigrum*, non-*Piper nigrum* and adulterant samples used in the study; Table S2: Validation results of the OPLS-DA models for the discrimination of *Piper nigrum* and non-*Piper nigrum* generated using the data from benchtop FTIR-ATR and FT-NIR and two handheld NIR spectrometers; Table S3: Validation results of the OPLS-DA models for the discrimination of black, white and red *Piper nigrum* generated using the data from benchtop FTIR-ATR and FT-NIR and two handheld NIR spectrometers.

## Abbreviations

The following abbreviations are used in this manuscript:

FTIR-ATR	Fourier Transform InfraRed-Attenuated Total Reflectance
FT-NIR	Fourier Transform Near-Infrared
OPLS-DA	Orthogonal Partial Least Squares-Discriminant Analysis
DD-SIMCA	Data-Driven Soft Independent Modelling of Class Analogy
DNA	DeoxyriboNucleic Acid
DART-MS	Direct Analysis in Real-Time-Mass Spectrometry
1H NMR	Proton Nuclear Magnetic Resonance
VIP	Variable Importance in Projection
PCA	Principal Component Analysis
ROC	Receiver Operating Characteristic
DRIFTS	Diffuse Reflectance Infrared Fourier Transform Spectroscopy
NIR-HSI	Near-Infrared Hyperspectral Imaging
