# Supplementary material for: Rapid Detection of Black Pepper Adulteration with Endogenous and Exogenous Materials: Assessment of Benchtop and Handheld Infrared Spectrometers
Source: Foods. 2026 Feb 19;15(4):754. doi: 10.3390/foods15040754 (PMC12939477; doi:10.3390/foods15040754)
Supplement: Supplementary file 1 [file foods-15-00754-s001.zip › Rentz et al_Supplementary Information.pdf]

Supplementary Information

TABLE S1. *Piper nigrum*, non-*Piper nigrum* and adulterant samples used in the study.

#	Class	Info	Country	Region
		Piper nigrum		
1	*Piper nigrum*	Black pepper	Mixed (India, Indonesia, Malaysia, Brazil, Vietnam, Cambodia, Tanzania)	Mixed (Asia, Africa, South America)
2	*Piper nigrum*	Tellicherry black pepper	India	Asia
3	*Piper nigrum*	Black pepper	Madagascar	Africa
4	*Piper nigrum*	Black pepper	Tanzania	Africa
5	*Piper nigrum*	Black pepper	Brazil	South America
6	*Piper nigrum*	Kampot black pepper PGI	Cambodia	Asia
7	*Piper nigrum*	Kampot red pepper PGI	Cambodia	Asia
8	*Piper nigrum*	Kampot white pepper PGI	Cambodia	Asia
9	*Piper nigrum*	Penja black pepper PGI	Cameroon	Africa
10	*Piper nigrum*	Penja white pepper PGI	Cameroon	Africa
11	*Piper nigrum*	Penia smoked white pepper PGI	Cameroon	Africa
12	*Piper nigrum*	Penia roasted white pepper PGI	Cameroon	Africa
13	*Piper nigrum*	Sarawak black pepper	Malaysia	Asia
14	*Piper nigrum*	Sarawak white pepper	Malaysia	Asia
15	*Piper nigrum*	Tellichery black pepper	India	Asia
16	*Piper nigrum*	Malabar black pepper	India	Asia
17	*Piper nigrum*	Malabar white pepper	India	Asia
18	*Piper nigrum*	Malabar roasted black pepper	India	Asia
19	*Piper nigrum*	Malabar dehydrated green pepper	India	Asia
20	*Piper nigrum*	Phu Quoc black pepper	Vietnam	Asia
21	*Piper nigrum*	Phu Quoc red pepper	Vietnam	Asia
22	*Piper nigrum*	Nam Bo black pepper	Vietnam	Asia
23	*Piper nigrum*	Nam Bo white pepper	Vietnam	Asia
24	*Piper nigrum*	Madagascar black pepper	Madagascar	Africa
25	*Piper nigrum*	Madagascar green pepper	Madagascar	Africa
26	*Piper nigrum*	Bahia black pepper	Brazil	South America
27	*Piper nigrum*	Bahia white pepper	Brazil	South America
28	*Piper nigrum*	Bahia red pepper	Brazil	South America
29	*Piper nigrum*	Lampong black pepper	Indonesia	Asia
30	*Piper nigrum*	Wayanad black pepper	India	Asia
31	*Piper nigrum*	Belem black pepper	Brazil	South America
32	*Piper nigrum*	Matale black pepper	Sri Lanka	Asia
33	*Piper nigrum*	Vanuatu black pepper	Vanuatu	Oceania
34	*Piper nigrum*	Kerala black pepper	India	Asia
35	*Piper nigrum*	Dak Lak red pepper	Vietnam	Asia
36	*Piper nigrum*	Sao Tomé white pepper	São Tomé and Príncipe	Africa
37	*Piper nigrum*	Bolovens red pepper	Laos	Asia
38	*Piper nigrum*	Putumayo black pepper	Colombia	South America
39	*Piper nigrum*	Kampot white pepper PGI	Cambodia	Asia
40	*Piper nigrum*	Kampot red pepper PGI	Cambodia	Asia
		**Non-*Piper nigrum***		
1	Non-*Piper nigrum*	Sichuan black pepper; *Zanthoxylum armatum*	Nepal	Asia
2	Non-*Piper nigrum*	Sichuan pepper; *Zanthoxylum piperitum*	China	Asia
3	Non-*Piper nigrum*	Sichuan pepper; *Zanthoxylum piperitum*	China	Asia
4	Non-*Piper nigrum*	Long red pepper Kampot; *Piper retrofractum*	Cambodia	Asia
5	Non-*Piper nigrum*	Long white Kampot pepper; *Piper retrofractum*	Cambodia	Asia
6	Non-*Piper nigrum*	Long black Kampot pepper; *Piper retrofractum*	Cambodia	Asia
7	Non-*Piper nigrum*	*Xylopia aethiopica*	Senegal	Africa
8	Non-*Piper nigrum*	Likouala pepper; *Piper guineense*	Congo	Africa
9	Non-*Piper nigrum*	Voatsiperifery red pepper; *Piper borbonense*	Madagascar	Africa
		**Adulterants**		

1	Adulterant	Black pepper spent	N/A	N/A
2	Adulterant	Pinheads	N/A	N/A
3	Adulterant	Papaya seeds	N/A	N/A
4	Adulterant	Red chilli pepper seeds	N/A	N/A
5	Adulterant	Red chilli pepper flesh	N/A	N/A
6	Adulterant	Walnut shells	N/A	N/A
7	Adulterant	Hazelnut shells	N/A	N/A
8	Adulterant	Millet	N/A	N/A
9	Adulterant	White sesame seeds	N/A	N/A
10	Adulterant	Buckwheat flour	N/A	N/A
11	Adulterant	White beans	N/A	N/A
12	Adulterant	Black lentils	N/A	N/A
13	Adulterant	Corn flour	N/A	N/A
14	Adulterant	Hemp seeds	N/A	N/A
15	Adulterant	Brown rice flour	N/A	N/A
16	Adulterant	Potato starch	N/A	N/A
17	Adulterant	Flaxseed	N/A	N/A
18	Adulterant	White sesame seeds	N/A	N/A
19	Adulterant	Yellow mustard seeds	N/A	N/A
20	Adulterant	White rice flour	N/A	N/A
21	Adulterant	Wheat flour	N/A	N/A
22	Adulterant	Corn starch	N/A	N/A
23	Adulterant	Cayenne pepper	N/A	N/A
24	Adulterant	Cayenne pepper	N/A	N/A
25	Adulterant	Olive pomace	N/A	N/A
26	Adulterant	Black mustard seeds	N/A	N/A
27	Adulterant	Green clay	N/A	N/A

N/A – Information not available

TABLE S2. Validation results of the OPLS-DA models for the discrimination of *Piper nigrum* and non-*Piper nigrum* generated using the data from benchtop FTIR-ATR and FT-NIR and two handheld NIR spectrometers.

**IR spectrometer**	**Type of spectrometer**	**OPLS-DA (n = 49)**		
		**R2X(cum)**	**R2Y(cum)**	**Q2(cum)**
Nicolet iS50 FTIR-ATR	benchtop	0.899	0.993	0.755
MPA II FT-NIR	benchtop	0.983	0.941	0.827
microNIR 1700ES	handheld	0.881	0.730	0.654
SCiO	handheld	0.998	0.466	0.350

TABLE S3. Validation results of the OPLS-DA models for the discrimination of black, white and red *Piper nigrum* generated using the data from benchtop FTIR-ATR and FT-NIR and two handheld NIR spectrometers.

**IR spectrometer**	**Type of spectrometer**	**OPLS-DA (n = 38) - black vs. white vs. red *P. nigrum***		
		**R2X(cum)**	**R2Y(cum)**	**Q2(cum)**
Nicolet iS50 FTIR-ATR	benchtop	0.888	0.893	0.717
MPA II FT-NIR	benchtop	0.992	0.876	0.757
microNIR 1700ES	handheld	0.998	0.901	0.803
SCiO	handheld	1.000	0.818	0.626
**IR spectrometer**	**Type of spectrometer**	**OPLS-DA (n = 32) - black vs. white *P. nigrum***		
		**R2X(cum)**	**R2Y(cum)**	**Q2(cum)**

Nicolet iS50 FTIR-ATR	benchtop	0.881	0.984	0.782
MPA II FT-NIR	benchtop	0.986	0.953	0.914
microNIR 1700ES	handheld	0.986	0.921	0.901
SCiO	handheld	1.000	0.939	0.921
**IR spectrometer**	**Type of spectrometer**	**OPLS-DA (n = 28) - black vs. red *P. nigrum***		
		**R2X(cum)**	**R2Y(cum)**	**Q2(cum)**
Nicolet iS50 FTIR-ATR	benchtop	0.768	0.927	0.697
MPA II FT-NIR	benchtop	0.978	0.873	0.718
microNIR 1700ES	handheld	0.990	0.852	0.808
SCiO	handheld	1.000	0.873	0.791
**IR spectrometer**	**Type of spectrometer**	**OPLS-DA (n = 16) - white vs. red *P. nigrum***		
		**R2X(cum)**	**R2Y(cum)**	**Q2(cum)**
Nicolet iS50 FTIR-ATR	benchtop	0.925	0.992	0.876
MPA II FT-NIR	benchtop	0.967	0.935	0.739
microNIR 1700ES	handheld	0.999	0.946	0.774
SCiO	handheld	1.000	0.833	0.710
